# Short-Term Solar Irradiance Prediction Based on Adaptive Extreme Learning Machine and Weather Data

**DOI:** 10.3390/s22218218

**Published:** 2022-10-27

**Authors:** Ahmad Alzahrani

**Affiliations:** Department of Electrical Engineering, College of Engineering, Najran University, Najran 11001, Saudi Arabia; asalzahrani@nu.edu.sa

**Keywords:** solar, irradiance, prediction, machine learning, adaptive ELM, Najran, GHI

## Abstract

Concerns over fossil fuels and depletable energy sources have motivated renewable energy sources utilization, such as solar photovoltaic (PV) power. Utilities have started penetrating the existing primary grid with renewable energy sources. However, penetrating the grid with photovoltaic energy sources degrades the stability of the whole system because photovoltaic power depends on solar irradiance, which is highly intermittent. This paper proposes a prediction method for non-stationary solar irradiance. The proposed method uses an adaptive extreme learning machine. The extreme learning machine method uses approximated sigmoid and hyper-tangent functions to ensure faster computational time and more straightforward microcontroller implementation. The proposed method is analyzed using the hourly weather data from a specific site at Najran University. The data are preprocessed, trained, tested, and validated. Several evaluation metrics, such as the root mean square error, mean square error, and mean absolute error, are used to evaluate and compare the proposed method with other recently introduced approaches. The results show that the proposed method can be used to predict solar irradiance with high accuracy, as the mean square error is 0.1727. The proposed approach is implemented using a solar irradiance sensor made of a PV cell, a temperature sensor, and a low-cost microcontroller.

## 1. Introduction

Renewable energy sources have the potential to satisfy the future energy demand because they are sustainable, clean, and cost effective. A study suggests that the path toward 100% of energy integration into the main grid is feasible in 2050 [1]. Several countries have already built large-scale renewable energy projects to gain economic advantages and reduce carbon footprint per capita [2]. The 300 MW Skaka solar PV project is launched in the first phase of the National Renewable Energy Program (NREP) in Saudi Arabia [3]. The project provides power to 44,000 houses and reduces carbon emissions by 606,000 tons/yr [4]. The second phase of the NREP includes seven solar PV projects with a total capacity of about 2.98 GW, and the fourth phase of NREP includes four solar PV projects with a full capacity of 1.2 GW. The Saudi Arabian 2030 vision aims to increase and diversify energy sources.

Solar PV energy is suitable for Saudi Arabia because of the high solar radiation and long average and peak hours [5]. However, residential solar PV in Saudi Arabia does not contribute significantly to the total energy supply as in Germany. The Electricity and Cogeneration Regulatory Authority (ECRA) introduced regulations for consumers who are eligible to install grid-tied PV systems [6], where all bill calculations and a net metering scheme are illustrated [7]. In a grid-tied PV system, the PV panels feed the electrical loads when solar irradiance is available. During nights and when no solar irradiance is available, the electric loads are fed by the main grid [8]. The connection of the PV panel to the main grid impacts the voltage stability of the system and leads to disturbances [9]. Therefore, several solutions have been introduced to mitigate the impact of penetrating the main grid [10], such as a meter data management system [11], smart transformers [12], smart inverters [13], an outage management system [14], and solar irradiance prediction [15]. Both grid-tied and off-grid PV systems can be improved if information about the future values of solar irradiance is accurately predicted [16]. That is, the charge controller will have enough information and enough time to decide to charge or discharge a battery or to run critical loads [17].

Several solar irradiance prediction methods have been introduced in the literature [18]. These methods are categorized based on either the prediction time horizon or the input data type [19]. So, based on the prediction time horizon, the prediction types are short term (e.g., five minutes ahead) or long term (e.g., one week ahead) [20]. The input of the predictors is historical data [21], meteorological data [22], or sky images [23]. Prediction solar irradiance models are classified into physical, statistical, and empirical models. In physical models, the GHI is modeled as a function of the tilt angle, DNI, or the DHI [24]. However, the error in simple physical model estimation is significant during cloudy days [25]. The empirical prediction models can be found in the literature and are often used to estimate monthly solar irradiance. The most widely used approach is a sunshine-based predictive model [26].

Statistical models are the most widely used for short-term solar irradiance [27]. Common statistical methods are autoregressive moving average (ARMA) [28], Hidden Markov Models (HMM) [29], and autoregressive integrated moving average (ARIMA) [30]. In these approaches, the Yule–Walker method is used to identify the coefficients [31], and before applying this approach, the time series should be tested for stationarity [32], which may be a disadvantage. Support vector regression (SVR) is the most extensively used approach to predict solar PV power [33]. The SVR algorithm is a supervised learning system. SVM is an effective technique for classification and prediction. SVRs are designed to construct decision boundaries using the notion of decision planes [34]. Although it has been employed in prediction, SVM may be incapable of extracting the time series’ long-term correlation or the extremely short-term components. Prediction based on an artificial neural network (ANN) [35] is used to relate the temperature and previous GHI values to predict the next value. Other studies use the convolutional neural network (CNN) by analyzing the spatiotemporal correlation of solar irradiance [36]. Most neural network-based approaches need training and backpropagation to update the weight and decrease the mean square error.

Therefore, new approaches, such as extreme learning machine, have been introduced to reduce the computation time and provide accurate results. However, the extreme learning machine does not update the output weight in each iteration. Therefore, an online update to output weight is needed. This paper presents an adaptive ELM approach implementation to predict solar irradiance and how to update its weight online. The proposed method differs from the original ELM in weight update and a buffer for the input values. The main contributions of this paper are as follows:

- It proposes two different approaches to solar irradiance prediction. These two approaches can predict irradiance with high accuracy and relatively less computational time.
- It presents a effective method for online adaptation of the output weight of the ELM method, which has less computational time.
- The developed models are trained, tested, and validated using local data with a 15 min/sample resolution.
- Implementation and testing of the adaptive ELM approach are carried out on a low-cost microcontroller.

The rest of this paper is structured and organized as follows: Section 2 presents site location and weather information, Section 3 presents some details about the theoretical analysis of the proposed approaches, and Section 4 presents the prediction process and the possibility of implementing low-cost hardware, and Section 5 presents the results of the proposed approach. Finally, the conclusion and future work are presented in Section 6.

## 2. Site Location and Data Acquirement

**Figure 1:**
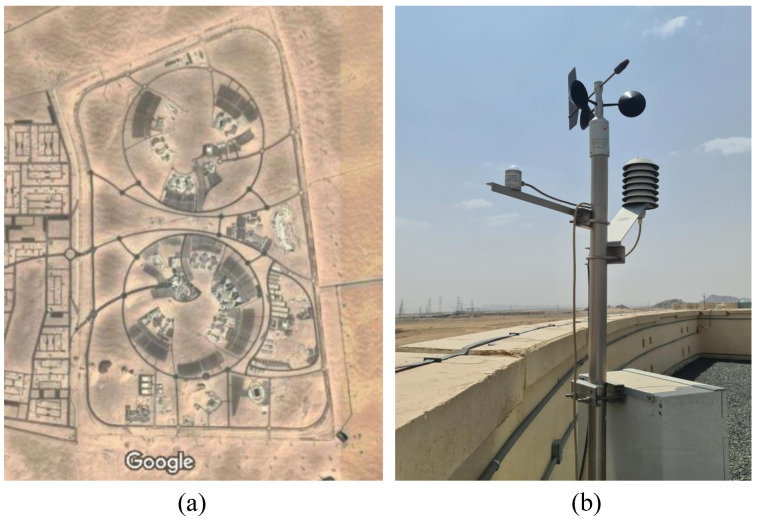
Site location used in this study. (**a**) Najran University; (**b**) Solar and wind weather station.

The site location used in this paper is Najran University in the southern part of the Kingdom of Saudi Arabia. The University is located at latitude 17.63228 North, longitude 44.53735 East, and elevation of 1290 m above sea level. Figure 1 shows the location of the Najran University and the weather station. The city’s climate and university surroundings are considered continental and relatively dry. Several data sources were found on the government website or weather websites. The data in this paper were obtained from Open data by K.A. Care, which contains monthly samples from 2014 to mid-2016. The hourly weather data can also be obtained from the National Solar Radiation Database (NSRDB) [37].

**Figure 2:**
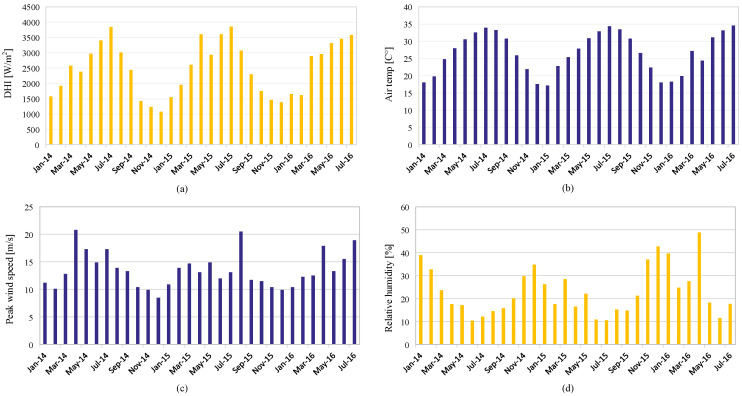
Monthly weather and diffused irradiance data of the Najran university. (**a**) The diffused horizontal irradiance. (**b**) Air temperature. (**c**) Peak wind speed. (**d**) Relative humidity.

**Figure 3:**
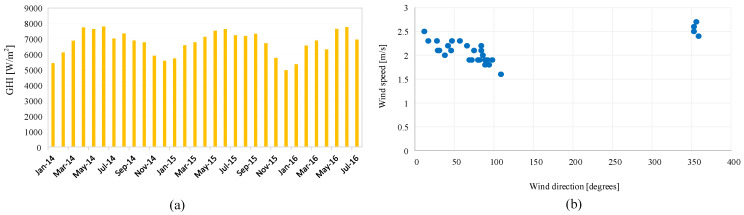
Normalization energy sources data at the Najran university. (**a**) The global horizontal irradiance in Wh/m${}^{2}$. (**b**) The average wind speed in [m/s] vs. wind direction.

**Figure 4:**
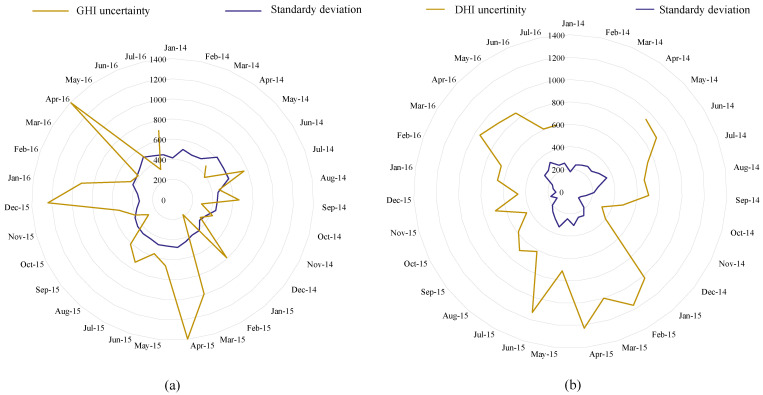
The standard deviation and uncertainty of the monthly (**a**) GHI (**b**) DHI.

Figure 2a shows the monthly average of the DHI, which indicates the amount of solar irradiance that does not arrive directly on the PV panel. The temperature of the air is shown in Figure 2b. The monthly average is between 15 C and 35 degrees. The peak wind speed is the highest in April and August of every year, as depicted in Figure 2c. The relative humidity data prove the continental climate, which is mostly less than 30%, as shown in Figure 2d. Although several parameters are included in the dataset, the most critical parameters in this study are the GHI, temperature, humidity, and wind speed. Global horizontal irradiance (GHI) is the total amount of solar radiation falling on a surface horizontal to the ground. Najran city has one of the highest monthly averages of GHI, as shown in Figure 3a. The average value of wind speed is shown in Figure 3b. The uncertainty and standard deviation of both GHI and DHI are shown in Figure 4.

## 3. Theoretical Illustration of Solar Irradiance Prediction Approaches

This section presents the theoretical background and mathematical formulation of the proposed prediction approaches.

### 3.1. Extreme Learning Machine

**Figure 5:**
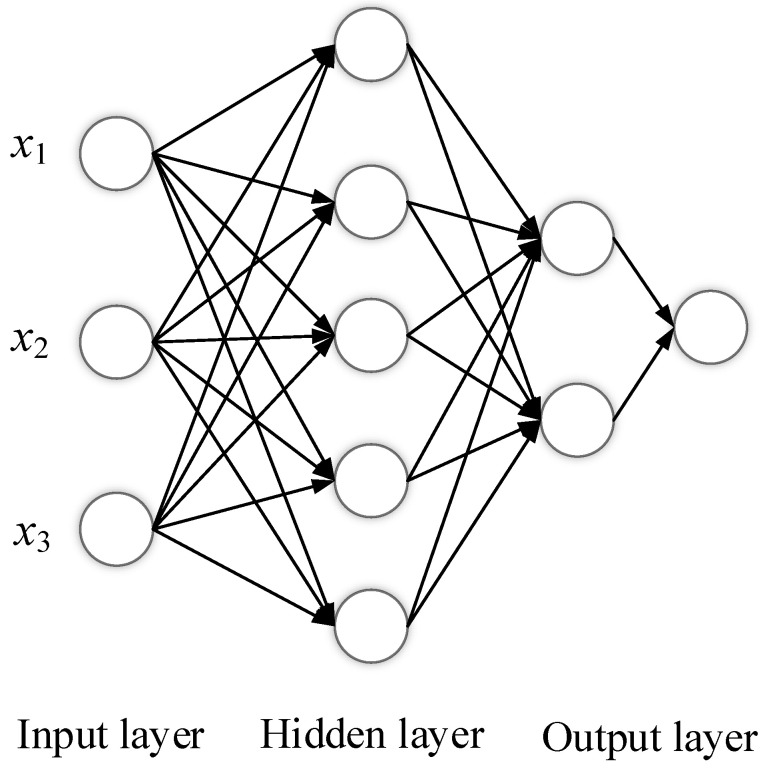
Single-layer extreme learning machine.

One major disadvantage of the feedforward backpropagation algorithm is its slow learning speed. Therefore, the extreme learning machine has been introduced to solve computation and speed issues. The ELM was introduced in 2006 by Huang [38] and was utilized in several applications ranging from speech recognition to image processing. Figure 5 shows a basic ELM network, where ELM is a feedforward neural network that has a single random hidden neurons layer. The main idea of this method is to find the weight vector that maps the output y to the transformed input *h*. The reason that ELM converges faster than the backpropagation algorithm is because of randomly hidden neurons. At the same time, ELM can have a better generalization and avoid overfitting issues. According to Bartlett’s theory, generalization performance is better when the training error is smaller. Therefore, ELM can reach the slightest training error and can run extremely quickly [9].

#### Random Hidden Nodes for SLFNs

Let us suppose that we have *N* arbitrary number of samples $\left(x_{i},y_{i}\right)$ where *x* is the input vector and $y_{i}$ is the output vector. The SLFNs with N hidden neurons can be mathematically modeled as follows:

(1) $$\sum_{i=1}^{N}\beta_{i}g_{i}\left(x_{j}\right)=\sum_{i=1}^{N}\beta_{i}g\left(w_{i}\cdot x_{i}+b_{i}\right)=y_{j}$$

where *w* is the weight vector that connects the neurons in the hidden neuron with input neurons, and β is the weight vector that connects the hidden neuron layer to the output layer. The term $\left(w_{i},\;x_{i}\right)$ represents the inner product $w_{i}$ and $x_{i}$. The *N* samples can be approximated with the zero error means that makes the sum of the difference between the predicted and actual value of the output equals zero, which is given by

(2) $$\sum_{j=1}^{N}∥y_{predicted}-y_{actual}∥=0$$

The ELM algorithm is a straightforward and efficient way to train the single hidden-layer neural network [39,40]. The algorithm of the ELM is illustrated in Algorithm 1.

**Algorithm 1** ELM algorithm
1:Given training data samples $\left(X_{i},t_{i}\right)$ and an activation function2:Assign random input bias and weight (b,w)3:Compute the output of the hidden layer4:Compute the weight matrix β


### 3.2. Adaptive Extreme Learning Machine

**Figure 6:**
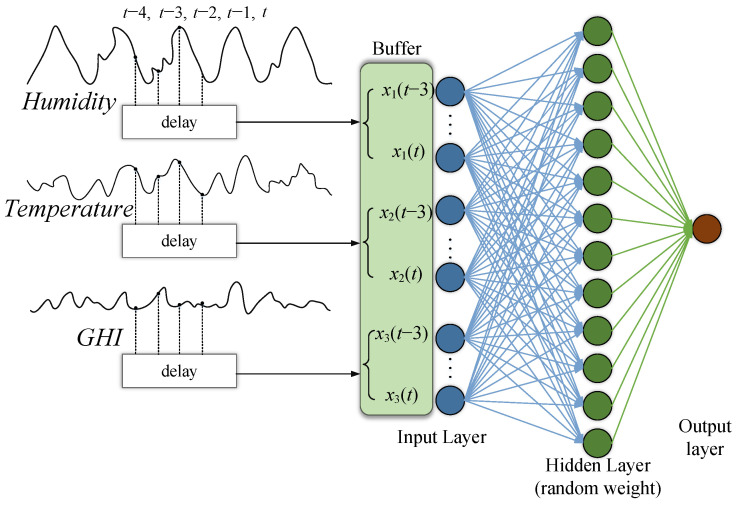
Extreme Learning Machine with time series input and sliding windows.

As mentioned previously, the simple aim for ELM is to calculate the output layer β that transforms the output of the hidden layer C to output Y. The figure shows the ELM with a buffer contains several previous samples from the input parameters. Next, we show the input

(3) $$\begin{array}{ccc} \; & x=\; & {\left[\mathrm{GHI}\;\mathrm{Temp}\;\mathrm{humidity}\right]}_{n\times z} \end{array}$$

where *z* is the number of input variables and *n* is the buffer size. The number of including previous samples determines the buffer size. The input layer is multiplied by a weight vector filled with random values, which is usually sampled from Gaussian random noise. Figure 6 shows the adaptive ELM used to predict the time series. The multiplication is mapped to the hidden layer through an activation function $G(a_{i},x)$

(4) $$G\left(a_{i},x\right)=\sigma \left(\begin{array}{cc} \; & {\left[\mathrm{GHI}\;\mathrm{Temp}\;\mathrm{humidity}\right]}_{n\times z} \end{array}{\left[\begin{array}{c} Normal-dist(\mu =0,\sigma^{2}=1)\\ \vdots \\ Normal-dist(\mu =0,\sigma^{2}=1) \end{array}\right]}_{z\times 1}\;\right).$$

The output from Equation (4) is transformed by hidden layer **C**, which is formulated as follows:

(5) $$C\left(a,x\right)={\left[\begin{array}{ccc} G(a_{1},x_{1}) & \cdots & G(a_{\tilde{N}},x_{1})\\ \vdots & \ddots & \vdots \\ G(a_{1},x_{n}) & \cdots & G(a_{\tilde{N}},x_{n}) \end{array}\right]}_{n\times \tilde{N}}$$

The matrix **C** is the output of the hidden neurons. The output **Y** is calculated by multiplying **C** with the output weight vector β, as follows:

(6) Y=Cβ

The aim is to predict the accurate value of solar irradiance, so the output weight matrix needs to be determined. The output weight matrix can be solved using the least square solution and can also be extended by a diagonal weight matrix.

(7) $$\beta ={\left(C^{⊤}C\right)}^{-1}C^{⊤}Y={\left(C^{⊤}WC\right)}^{-1}C^{⊤}WY\;.$$

In light of newly generated sample data pairs, dividing the solution into offline and online components would make it possible to reduce the need for processing power and storage space. In order to do this, the matrices are first decomposed into an offline part and an online part, which can be represented by:

(8) $$\begin{array}{c} C={\left[\begin{array}{c} C_{\mathrm{OF}}\\ C_{\mathrm{ON}} \end{array}\right]}_{n\times \tilde{N}}\;W={\left[\begin{array}{cc} W_{\mathrm{OF}} & 0\\ 0 & W_{\mathrm{ON}} \end{array}\right]}_{n\times N}\;Y={\left[\begin{array}{c} Y_{\mathrm{OF}}\\ Y_{\mathrm{ON}} \end{array}\right]}_{n\times 1}\;. \end{array}$$

The derivation for the recursive least square method is quite similar to that of the feedforward ELM, except it includes an additional weight to inversion part $C^{⊤}WC$. The inversion part is also denoted offline and online by $C^{⊤}WC$, respectively. Therefore, the inverted part of Equation (7) is simplified to:

(9) $$\begin{array}{cc} \; & K_{\mathrm{ON}}=C^{⊤}WC=\left[\begin{array}{cc} C_{\mathrm{OF}}^{⊤} & C_{\mathrm{ON}}^{⊤} \end{array}\right]\left[\begin{array}{cc} W_{\mathrm{OF}} & 0\\ 0 & W_{\mathrm{ON}} \end{array}\right]\left[\begin{array}{c} C_{\mathrm{OF}}\\ C_{\mathrm{ON}} \end{array}\right]\\ \; & =C_{\mathrm{OF}}^{⊤}W_{\mathrm{OF}}C_{\mathrm{OF}}+C_{\mathrm{ON}}^{⊤}W_{\mathrm{ON}}C_{\mathrm{ON}}=K_{\mathrm{OF}}+C_{\mathrm{ON}}^{⊤}W_{\mathrm{ON}}C_{\mathrm{ON}}\;. \end{array}$$

Similarly, the non-inverted part of Equation (7) can be simplified to:

(10) $$\begin{array}{cc} \; & C^{⊤}WY=\left[\begin{array}{cc} C_{\mathrm{OF}}^{⊤} & C_{\mathrm{ON}}^{⊤} \end{array}\right]\left[\begin{array}{cc} W_{\mathrm{OF}} & 0\\ 0 & W_{\mathrm{ON}} \end{array}\right]\left[\begin{array}{c} Y_{\mathrm{OF}}\\ Y_{\mathrm{ON}} \end{array}\right]=C_{\mathrm{OF}}^{⊤}W_{\mathrm{OF}}Y_{\mathrm{OF}}+C_{\mathrm{ON}}^{⊤}W_{\mathrm{ON}}Y_{\mathrm{ON}}\\ & \;\;\;\;\;\;\;\;\;\;\;\;=K_{\mathrm{OF}}K_{\mathrm{OF}}^{-1}C_{\mathrm{OF}}^{⊤}W_{\mathrm{OF}}Y_{\mathrm{OF}}+C_{\mathrm{ON}}^{⊤}W_{\mathrm{ON}}Y_{\mathrm{ON}}\\ & \;\;\;\;\;\;\;\;\;\;\;\;=K_{\mathrm{OF}}\beta_{\mathrm{OF}}+C_{\mathrm{ON}}^{⊤}W_{\mathrm{ON}}Y_{\mathrm{ON}}\\ & \;\;\;\;\;\;\;\;\;\;\;\;=\left(K_{\mathrm{ON}}-C_{\mathrm{ON}}^{⊤}W_{\mathrm{ON}}C_{\mathrm{ON}}\right)\beta_{\mathrm{OF}}+C_{\mathrm{ON}}^{⊤}W_{\mathrm{ON}}Y_{\mathrm{ON}}\\ & \;\;\;\;\;\;\;\;\;\;\;\;=K_{\mathrm{ON}}\beta_{\mathrm{OF}}-C_{\mathrm{ON}}^{⊤}W_{\mathrm{ON}}C_{\mathrm{ON}}\beta_{\mathrm{OF}}+C_{\mathrm{ON}}^{⊤}W_{\mathrm{ON}}Y_{\mathrm{ON}}\;. \end{array}$$

Using Equation (10) to obtain the full online weight

(11) $$\begin{array}{cc} \; & \beta_{\mathrm{ON}}=\left(K_{\mathrm{ON}}^{-1}\right)\left(C^{⊤}WY\right)=\beta_{\mathrm{OF}}-K_{\mathrm{ON}}^{-1}C_{\mathrm{ON}}^{⊤}W_{\mathrm{ON}}C_{\mathrm{ON}}\beta_{\mathrm{OF}}+K_{\mathrm{ON}}^{-1}C_{\mathrm{ON}}^{⊤}W_{\mathrm{ON}}Y_{\mathrm{ON}}\\ & \;\;\;\;=\beta_{\mathrm{OF}}+K_{\mathrm{ON}}^{-1}C_{\mathrm{ON}}^{⊤}W_{\mathrm{ON}}\left(Y_{\mathrm{ON}}-C_{\mathrm{ON}}\beta_{\mathrm{OF}}\right) \end{array}$$

The online weight can be calculated without the requirement for the offline dataset. The $\tilde{N}\times \tilde{N}$ sized K inverse can be computationally expensive. In order to reduce the computations, a smaller-sized buffer for the previous value of the solar irradiance is used. One can let $P=K^{-1}$

(12) $$\begin{array}{cc} P_{\mathrm{OF}} & =K_{\mathrm{OF}}^{-1}={\left(C_{\mathrm{OF}}^{⊤}W_{\mathrm{OF}}C_{\mathrm{OF}}\right)}^{-1} \end{array}$$

(13) $$\begin{array}{cc} P_{\mathrm{ON}} & =K_{\mathrm{ON}}^{-1}={\left(P_{\mathrm{OF}}^{-1}+C_{\mathrm{ON}}^{⊤}W_{\mathrm{ON}}C_{\mathrm{ON}}\right)}^{-1} \end{array}$$

and apply the Woodbury formula on Equation (13) to get the following:

(14) $$\begin{array}{c} P_{\mathrm{ON}}=P_{\mathrm{OF}}-P_{\mathrm{OF}}C_{\mathrm{ON}}^{⊤}{\left(W_{\mathrm{ON}}^{-1}+C_{\mathrm{ON}}P_{\mathrm{OF}}C_{\mathrm{ON}}^{⊤}\right)}^{-1}C_{\mathrm{ON}}P_{\mathrm{OF}}\;. \end{array}$$

More simplification can be applied to the presented algorithm, but first, the term the $C_{\mathrm{ON}}^{⊤}W_{\mathrm{ON}}$ part is added to leaves the term of Equation (11) to Equation (14) and distributes $C_{\mathrm{ON}}^{⊤}$ to get the following equation

(15) $$\begin{array}{c} P_{\mathrm{ON}}C_{\mathrm{ON}}^{⊤}W_{\mathrm{ON}}=\left[P_{\mathrm{OF}}C_{\mathrm{ON}}^{⊤}-P_{\mathrm{OF}}C_{\mathrm{ON}}^{⊤}{\left(W_{\mathrm{ON}}^{-1}+C_{\mathrm{ON}}P_{\mathrm{OF}}C_{\mathrm{ON}}^{⊤}\right)}^{-1}\cdot C_{\mathrm{ON}}P_{\mathrm{OF}}C_{\mathrm{ON}}^{⊤}\right]W_{\mathrm{ON}}\;. \end{array}$$

Equation (15) is clarified by substituting $A=P_{\mathrm{OF}}C_{\mathrm{ON}}^{⊤}$, $B=C_{\mathrm{ON}}A$ and then distributing $W_{\mathrm{ON}}$ to obtain the following equation:

(16) $$\begin{array}{c} P_{\mathrm{ON}}C_{\mathrm{ON}}^{⊤}W_{\mathrm{ON}}=A\left[W_{\mathrm{ON}}-{\left(W_{\mathrm{ON}}^{-1}+B\right)}^{-1}BW_{\mathrm{ON}}\right]\;. \end{array}$$

After further simplification, the following equation

(17) $$P_{\mathrm{ON}}C_{\mathrm{ON}}^{⊤}W_{\mathrm{ON}}=A{\left(W_{\mathrm{ON}}^{-1}+B\right)}^{-1}\;.$$

The inverse relation between $P_{\mathrm{ON}}$ and $K_{\mathrm{ON}}^{-1}$ is known. Therefore, $P_{\mathrm{ON}}C_{\mathrm{ON}}^{⊤}W_{\mathrm{ON}}=K_{\mathrm{ON}}^{-1}C_{\mathrm{ON}}^{⊤}W_{\mathrm{ON}}$. The online output weight can be solved by substituting Equation (17) into Equation (11). The summary of the offline and online computation is illustrated in Table 1.

### 3.3. Feed Forward Neural Network Based Particle Optimization

**Figure 7:**
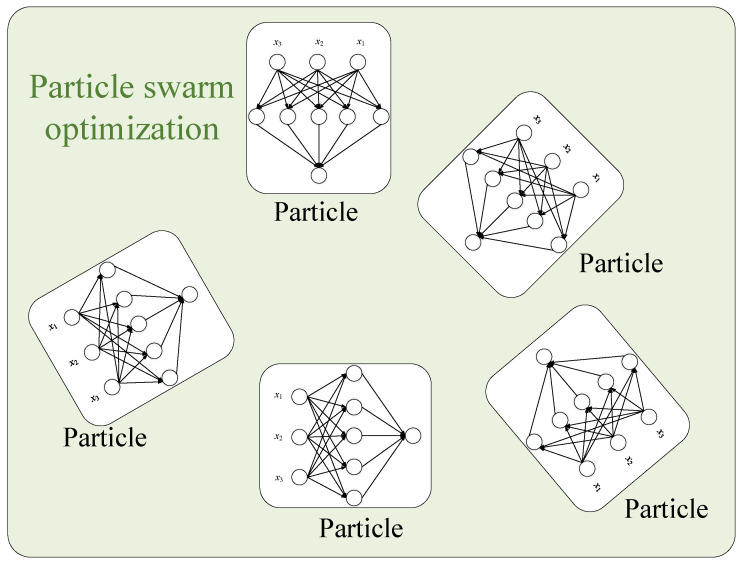
Feed forward neural network trained with PSO.

This is similar to the conventional particle swarm optimization algorithm. However, the particles are the neural network’s output, as shown in Figure 7. The algorithm completes the search when the optimal weight is calculated. A similar approach is used in energy-management systems with the Internet of Things (IoTs) [41], and in the prediction of the composite behavior [42].

## 4. Prediction Methodology

**Figure 8:**
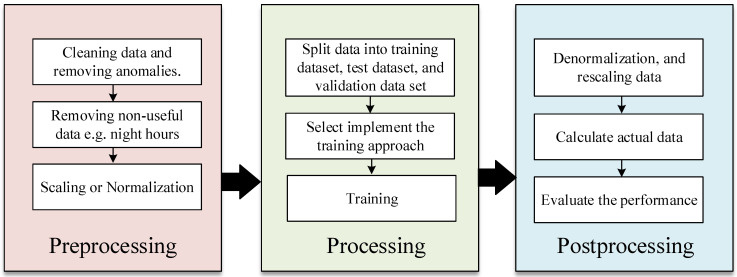
The prediction methodology, which consists of three stages.

The prediction process is divided into three stages, as shown in Figure 8. The first stage is preprocessing, where the data are prepared; the second stage is about training a machine learning model. The last stage is the post-processing and the performance evaluation.

### 4.1. Data Preprocessing and Data Cleaning

**Figure 9:**
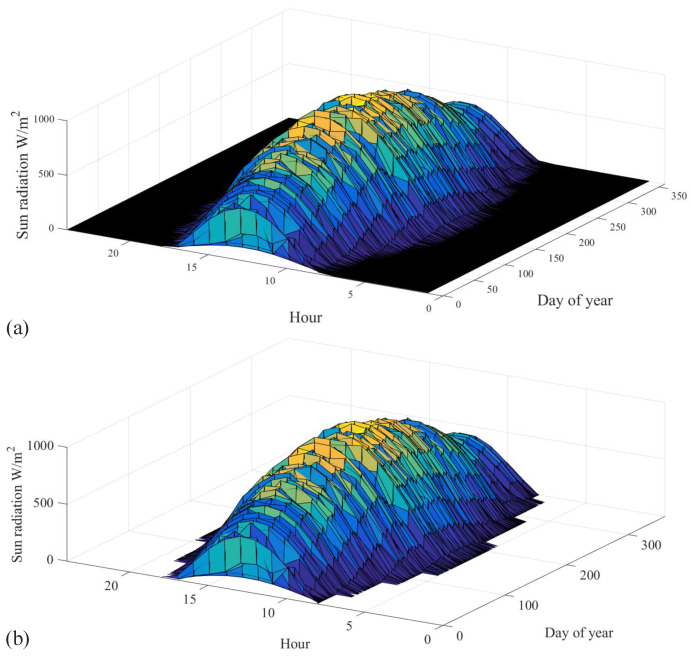
The data cleaning process (**a**) the data before eliminating night hours (**b**) the data after eliminating night hours.

The dataset represents three years of weather samples; each year contains 17,520 samples. Raw data often contain errors and misreadings, which require preprocessing before they can be used in model training. The first step is to clean the data from anomalous sensor readings and missing data points with interpolated values. An example of incorrect readings is when the GHI has negative values, where the minimum value of solar irradiance is zero watts per square meter. Advanced techniques can also be used to clean the data, such as incorporating maximum temperature and humidity change per day. After cleaning the data, only valuable data and features are used. The useful data are the data points that start from 6 a.m. to 8 p.m., only during the day. The season affects the sunrise and sunset time, and the same goes for the peak hours. Figure 9a illustrates the irradiance before removing irradiance, and Figure 9b shows the irradiance after getting rid of non-useful irradiance data.

After cleaning the data and extracting valuable information, normalization is applied to scale all data between two values. Normalization allows the model to be trained faster, helps us avoid being trapped in local minima, and makes the gradient-based algorithms treat all features equally. The normalization used in this study is between zero and one, and the following equation is used to normalize the data.

(18) $$\tilde{\mathrm{GHI}}=\frac{\mathrm{GHI}-{\mathrm{GHI}}_{\min}}{{\mathrm{GHI}}_{\max}-{\mathrm{GHI}}_{\min}}$$

where ${\mathrm{GHI}}_{\min}$ and ${\mathrm{GHI}}_{\max}$ are the minimum and the maximum values of the original GHI dataset, respectively, and GHI and $\tilde{\mathrm{GHI}}$ are the normalized and the denormalized value of the solar irradiance, respectively. Similarly, the temperature and humidity follow the same normalization process.

### 4.2. Processing Stage

After preprocessing, the data are split into training, testing, and validation sets. The training takes a major portion of the data, accounting for about 70% of the dataset. The testing and validation account for 15% each. The adaptive ELM model begins by populating the weight matrices with random numbers. The random numbers are used in high precision to avoid the singularity. Then, the initial hidden layer matrix **C** is calculated based on previous samples. Then, the buffer size is selected. It is usually selected based on trial and error. Then, the computation initial training is completed using the equations listed in Table 1. The online adaptive mode only needs initial training once, and there is no need for previous results after that. The output weight matrix in online mode is calculated, and then the next value of GHI can be calculated. Note that implementation of this method on low-cost hardware depends on the complexity of the activation functions.

**Figure 10:**
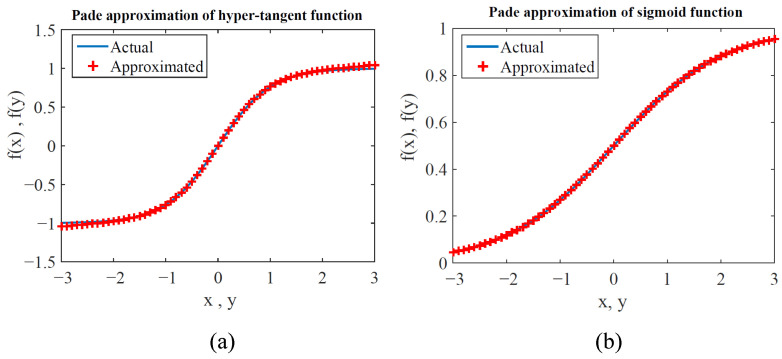
Padé approximation of the activation function. (**a**) Tanh activation function. (**b**) Sigmoid function.

The activation function brings a degree of nonlinearity to the data, and several types of activation functions use the exponent function. The most common are logistic function and hyperbolic tangent activation function, and they are given in (19) and (20), respectively. Note that some microcontrollers and digital signal processing devices will be significantly slowed if assigned to calculate the exponential function. Therefore, an approximation of the activation function can be used in this case to speed up the computation. Using Padé approximation, the logistic and hyperbolic tangent are given in (21) and (22), respectively. Figure 10 approximated functions fit well for the range from [−1, 1] and where the activation function is used to form the network [43].

(19) $$sigmoid\left(x\right)=\frac{1}{1+e^{-x}}$$

(20) $$tanh\left(x\right)=\frac{e^{x}-e^{-x}}{e^{x}+e^{-x}}$$

(21) $$f{\left(y\right)}_{sigmoid}=\frac{120+60y+12y^{2}+y^{3}}{120-60y+12y^{2}-y^{3}}$$

(22) $$f{\left(y\right)}_{tanh}=\frac{y(y^{2}+15)}{6y^{2}+15}$$

### 4.3. Post Processing Stage

The first step after we found the predicted values was to denormalize the data. Data can be renormalized using the following equation

(23) $$\mathrm{GHI}=\left(\frac{\tilde{\mathrm{GHI}}-{\tilde{\mathrm{GHI}}}_{\min}}{{\tilde{\mathrm{GHI}}}_{\max}-{\tilde{\mathrm{GHI}}}_{\min}}\right)$$

where $\tilde{{\mathrm{GHI}}_{\min}}$ and $\tilde{{\mathrm{GHI}}_{\max}}$ are the minimum and the maximum values of the normalized GHI dataset, respectively. The GHI and $\tilde{\mathrm{GHI}}$ are the normalized and the denormalized value of the solar irradiance, respectively. Similarly, the temperature and humidity follow the same denormalization process. The data are checked to see if there are no outliers or negative values resulting from ill-conditioned matrices. The performance of a model can be evaluated using various methods, such as the mean absolute error (MAE), the mean squared error (MSE), and the root mean squared error (RMSE). They can be calculated using (24)–(26), respectively. In hardware implementation and testing, only MSE is used, which determines the quality of the predictor.

(24) $$\mathrm{MAE}=\frac{1}{n}\sum_{i=1}^{n}\left|{\mathrm{GHI}}_{\mathrm{predicted}}\left[i\right]-{\mathrm{GHI}}_{\mathrm{actual}}\left[i\right]\right|$$

(25) $$\mathrm{MSE}=\frac{1}{n}\sum_{i=1}^{n}{\left({\mathrm{GHI}}_{\mathrm{predicted}}\left[i\right]-{\mathrm{GHI}}_{\mathrm{actual}}\left[i\right]\right)}^{2}$$

(26) $$\mathrm{RMSE}=\sqrt{\frac{1}{n}\sum_{i=1}^{n}{\left({\mathrm{GHI}}_{\mathrm{predicted}}\left[i\right]-{\mathrm{GHI}}_{\mathrm{actual}}\left[i\right]\right)}^{2}}$$

where *n* is the number of samples, $GHI_{actual}$ is the real values, and $GHI_{predicted}$ is the output values of the model.

## 5. Results and Discussion

**Figure 11:**
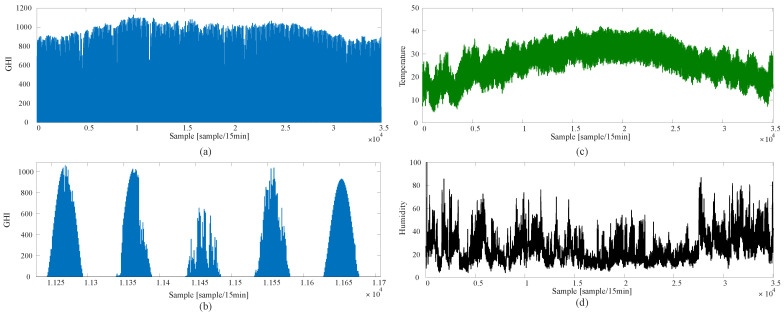
The data used in training and testing. (**a**) The GHI for a whole year. (**b**) The GHI for five working days. (**c**) The temperature in degree celsius. (**d**) Relative humidity in percentage.

**Figure 12:**
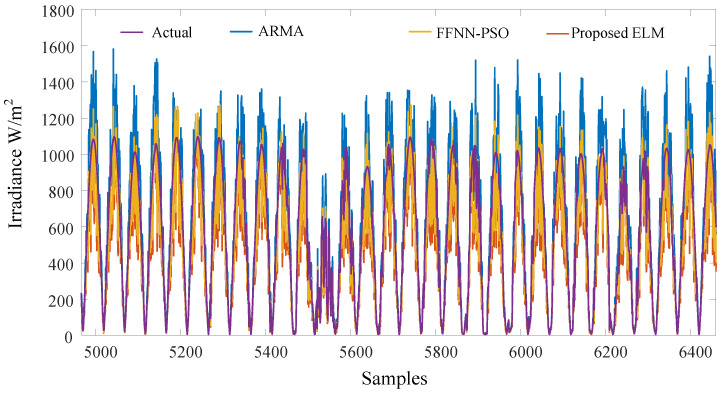
The predicted samples of the ARMA, FNN-PSO, and the proposed method compared to the actual values of GHI.

**Figure 13:**
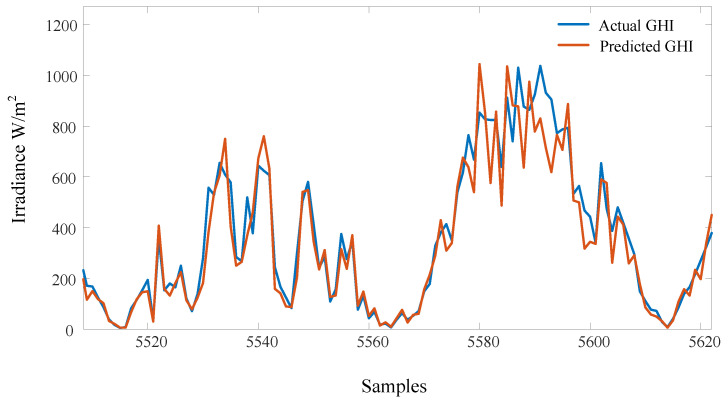
The predicted samples of the proposed method compared to the actual values of GHI. The samples are recorded every 15 min.

**Figure 14:**
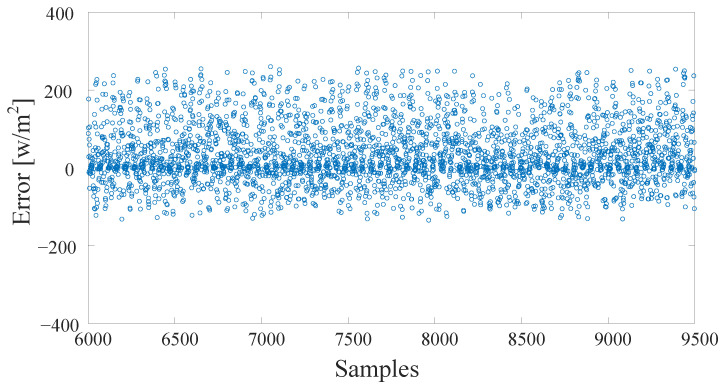
The error in samples prediction of the GHI, which is the difference between the proposed method’s output and the GHI’s actual values. Note that the samples are concentrated near 0, and the maximum error is less than 250 $\frac{W}{m^{2}}$.

The proposed algorithm is compared to several commonly used approaches, such as ARMA and FFNN-based PSO. Figure 11 shows the samples of the data used in training and testing. Each sample was taken 15 min after the previous one. The irradiance is less than 1150 W/m${}^{2}$, the maximum temperature is less than 43 degrees, and the humidity is mostly less than 50%. Figure 12 shows the overall performance of the algorithm. The ARMA has the fastest training time. However, it shows the worst performance, since the predicted output significantly differs from the actual values, especially during peak hours. The FFNN-PSO is an enhanced performance over the ARMA, but it takes a very long time to train and test, and still, there are some significant mismatching results. The proposed approach produces better results and requires less computation time compared to the other techniques, as the predicted values are close to the actual values. The performance of the previously mentioned algorithms is compared in terms of MAE, MSE, and RMSE, as shown in Table 2. The ARMA approach has the worst performance as the MAE is 0.3124, MSE is 0.233, and RMSE is 0.4463. MAE of about 0.2675 indicates the FFNN-PSO performance, MSE of 0.1880, and 0.3684 RMSE. The proposed method performs slightly better, where the MAE is 0.2444, MSE is 0.1727, and RMSE is 0.3012. The proposed method’s predicted samples are shown in Figure 13. The error, which is the absolute error, is shown in Figure 14.

**Figure 15:**
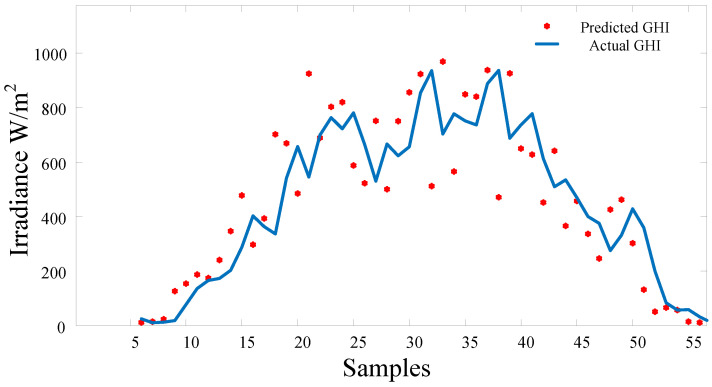
The experimental results of GHI. The predicted results (dots) are read every 15 min and compared to the actual GHI (solid line).

The adaptive ELM and FFNN-PSO are used as predictive approaches in hardware implementation (Atmega328 and PC). The results are presented and compared to a linear regression algorithm, as listed in Table 3. Different prediction times were experimented with to show the correlation between the predicted value and the previous hours or time horizon. It can be noticed that prediction in shorter time horizons yields better results. Therefore, the trade-off between computation time and time horizon must be figured out to obtain the optimized results. The adaptive ELM is implemented in real time to examine the performance. Data were collected from 6:30 a.m. until 5:30 p.m. on 18 August 2022. Figure 15 are samples of the graphs of data taken at a resolution of a single data point per 15 min. Data were acquired on the south side of the Engineering College building at Najran University. The results show the robust performance and ability to estimate the next value of solar irradiance.

## 6. Conclusions

This paper presented a prediction approach based on an adaptive extreme learning machine. The proposed algorithm works both offline and online, with reduced computational time and higher prediction accuracy. The dataset used in this study is based on a local site at Najran University. Several preprocessing steps were taken to ensure the training data are valuable, clean, and suitable for training predictive models. The offline performance of the proposed approach yields the lowest mean square error, about 0.1727, and the lowest mean absolute error, less than 0.25. Furthermore, the presented method can be implemented on hardware, which can be tested in less than 0.0062s, with a mean square error of about 0.2459. In the future, this method can be integrated into the whole energy management system or utilities to enhance the performance of the power grid and allow a higher level of renewable energy integration.

## Figures and Tables

**Table 1:** Summary of the offline and online computations.

Stage	Equations
Initial Training (offline)	$\begin{array}{c} P_{\mathrm{OF}}={\left[{\left(C_{\mathrm{OF}}^{⊤}W_{\mathrm{OF}}C_{\mathrm{OF}}\right)}^{-1}\right]}_{\tilde{N}\times \tilde{N}}\\ \beta_{\mathrm{OF}}={\left[P_{\mathrm{OF}}C_{\mathrm{OF}}^{⊤}W_{\mathrm{OF}}Y_{\mathrm{OF}}\right]}_{\tilde{N}\times 1} \end{array}$
Online Adaptive mode	$\begin{array}{c} A=P_{\mathrm{OF}}C_{\mathrm{ON}}^{⊤},\;B=C_{\mathrm{ON}}A\\ \beta_{\mathrm{ON}}=\beta_{\mathrm{OF}}+A{\left(W_{\mathrm{ON}}^{-1}+B\right)}^{-1}\left(Y_{\mathrm{ON}}-C_{\mathrm{ON}}\beta_{\mathrm{OF}}\right) \end{array}$
Online Prediction	$GHI=Y_{n+1}=C\left(a,x_{n+1}\right)\beta_{\mathrm{ON}}$

**Table 2:** Performance comparison between different algorithms.

Prediction Approach	MAE	MSE	RMSE
ARMA	0.3124	0.2133	0.4463
FFNN-PSO	0.2675	0.1880	0.3684
Proposed method	0.2444	0.1727	0.3012

**Table 3:** Comparison of the experimental performance.

Method	Training Time	Training MSE	Testing Time	Testing MSE
Initial ELM	0.0252	-	0.0046	0.2511
Adaptive ELM	0.2884	-	0.0062	0.2459
1 h regression	0.0022	0.3262	0.0007	0.4257
2 h regression	0.0094	0.4144	0.0005	0.4282
1 h NN PSO	30.5079	0.2320	0.0912	0.2552
4 h NN PSO	43.9875	0.1679	0.0020	0.2024

## Data Availability

Data could be shared upon request.

## Abbreviations

The following abbreviations are used in this manuscript:

ELM	Extreme learning machine.
FFNN	Feedforward neural network.
GHI	Global horizontal irradiance.
DNI	Direct Normal Irradiance letter acronym.
PSO	Particle swarm optimization.
NSRDB	The National Solar Radiation Database.
